# 

**Published:** 1891-08

**Authors:** 


### CONTENTS.

ORIGINAL COMMUNICATIONS—

THE TREATMENT OF ORGANIC STRICTURE OF THE UERTHRA BY COMBINED INTERNAL AND EXTERNAL URETHROTOMY, WITH PERINEAL DRAINAGE. By F. W. McRae, M. D., Atlanta, Ga. .... 321

STERILITY IN WOMAN—ITS ÆTIOLOGY AND TREATMENT. By E. S. McKee, M. D., Cincinnati, Ohio .... 326

FIFTY YEARS' EXPERIENCE IN OBSTETRICS. By John S. Clark, M. D., Chicago, Ill. .... 333

CONCENTRATED FOOD IN THE TREATMENT OF PULMONARY CONSUMPTION. By Thos. J. Mays, M. D., Philadelphia, Pa. .... 346

ONE HUNDRED CONSECUTIVE CASES OF SKIN DISEASE. By M. B. Hutchins, M. D., Atlanta, Ga. .... 349

CORRESPONDENCE—

OUR NEW YORK LETTER .... 354

LONDON LETTER .... 359

EDITORIAL—

THE LEGISLATURE *vs.* THE PHYSICIANS .... 366

IN MEMORY OF DR JOS. P. LOGAN .... 368

ITEMS .... 353, 365, 370

SELECTIONS .... 371 to 381

BOOK REVIEWS .... 382

GEORGIA MEDICAL COLLEGE AGREEMENT .... 383

